# The Dreadnought and the Explosion

**Published:** 1917-02-03

**Authors:** 


### THE DREADNOUGHT AND THE EXPLOSION.

As is well known, the Seamen's Hospital Society has a branch at the Albert Docks, which had to bear the brunt of the work of attending to the injured immediately and for some time after the explosion.

The explosion took place shortly after 7 P.M. on the night of January 19, and the victims began to arrive at this branch in cabs, lorries, carts, and every other available conveyance. So fast did they arrive that all the wards, the hall, and the forecourts were soon full of stretchers bearing the injured, a long line extending far out on to the pavement.

There were at the hospital two doctors, the matron, three sisters, and fourteen nurses; unfortunately, the electric light had been put out of action and everything had to be done by the light of candles; this, together with the crowded state of the wards, made the doctors' and nurses' work most difficult.

With the limited number of medical men, the limited number of nurses, and the limited light it was seen that it would be impossible to treat the whole, or even half the cases that presented themselves. As everyone was fully occupied inside the hospital great difficulty arose outside, as there was no one to take charge and direct. However, after some delay various officials appeared on the scene, and the overflow was dispatched as quickly as possible to other hospitals after the cases had received first-aid.

Notwithstanding the difficulties, the work went on so well that by 10 P.M. 200 patients had been treated, a large number of whom were accommodated in the wards. Of these, seven died, in addition to three who died before reaching the wards.

Considerable damage was done to this branch's buildings. The glass throughout was shattered and some buildings were brought down. This naturally added greatly to the suffering of those who were being treated.

At the parent hospital at Greenwich forty-nine injured were treated, of whom three were retained in the wards. Here also considerable damage was done, glass being broken, window frames blown out, and ceilings brought down.

For some time past both the hospital at Greenwich and the branch at the Albert Dock have had their accommodation strained to the utmost limit. This is partly due to the fact that during the war the Society has received ratings from the Royal Navy, indeed last year they received no less than 1,165 men sent in from the different naval depôts.

